# Socioeconomic inequalities in self-assessed health and food consumption: the mediating roles of daily hassles and the perceived importance of health

**DOI:** 10.1186/s12889-023-15077-0

**Published:** 2023-03-07

**Authors:** Sanne E. Verra, Maartje P. Poelman, Andrea L. Mudd, Emely de Vet, John de Wit, Carlijn B. M. Kamphuis

**Affiliations:** 1 Department of Interdisciplinary Social Science, Utrecht University, Padualaan 14, 3584 CH Utrecht, the Netherlands; 2 Chair Group Consumption and Healthy Lifestyles, Wageningen University & Research, Hollandseweg 1, 6706 KN Wageningen, the Netherlands

**Keywords:** [MeSH terms]: socioeconomic factors, Health status disparities, Food intake

## Abstract

**Background:**

Urgent daily hassles, which are more common among people with a lower socioeconomic position (SEP), might limit one’s ability to address less pressing goals, such as goals related to health promotion. Consequently, health goals may be viewed as less focal, which could jeopardize one’s health. This study examined an understudied pathway: whether a higher severity of daily hassles resulted in a lower perceived importance of health and whether these two factors sequentially mediate socioeconomic inequalities in self-assessed health (SAH) and food consumption.

**Methods:**

A cross-sectional survey among 1,330 Dutch adults was conducted in 2019. Participants self-reported SEP (household income, educational level), the severity of eleven daily hassles (e.g., financial hassles, legal hassles), the perceived importance of health (not being ill, living a long life), SAH, and food consumption. Structural equation modeling was used to examine whether daily hassles and the perceived importance of health sequentially mediated income and educational inequalities in SAH, fruit and vegetable consumption (FVC) and snack consumption.

**Results:**

No evidence of sequential mediation through daily hassles and the perceived importance of health was found. Daily hassles individually mediated income inequalities in SAH (indirect effect: 0.04, total effect: 0.06) and in FVC (indirect effect: 0.02, total effect: 0.09). The perceived importance of not being ill and living a long life both individually mediated educational inequalities in SAH (indirect effects: 0.01 and -0.01, respectively, total effect: 0.07).

**Conclusions:**

Income inequalities in SAH and FVC were explained by daily hassles, and educational inequalities in SAH were explained by the perceived importance of health. Socioeconomic inequalities may not be sequentially explained by a more severe experience of daily hassles and a lower perceived importance of health. Interventions and policies addressing challenging circumstances associated with a low income may improve SAH and healthy food consumption among lower-income groups.

**Supplementary Information:**

The online version contains supplementary material available at 10.1186/s12889-023-15077-0.

## Background

Socioeconomic inequalities in health and health behaviors persist worldwide [1]. Those with a low socioeconomic position (SEP) spend fewer years of their lives in good health and die at an earlier age than those with a higher SEP [2]. In the Netherlands, people with a higher SEP spend, on average, eighteen more years of their lives in good health than those with a lower SEP [3]. Many mechanisms explain these inequalities, including inequalities in healthy food consumption [2]. People with a low SEP have a lower fruit and vegetable consumption (FVC), and a higher snack consumption compared to people with a high SEP [4, 5]. These consumption patterns increase the risk of diet-related non-communicable diseases, such as cardiovascular diseases, type 2 diabetes, and cancer [2]. Investigations into potential explanatory pathways are needed to inform the development of policies and interventions to reduce inequalities in health and healthy food consumption.

Socioeconomic inequalities in health and food consumption may be partially explained by a socioeconomic gradient in the experience of daily hassles [6, 7]. The life stress hypothesis suggests that, because of challenging day-to-day circumstances, those with a lower SEP are disproportionally affected by a wide range of acute and chronic daily hassles compared to those with a higher SEP [8], including hassles of structural and psychosocial origins [9–11]. Examples of daily hassles of a structural origin include those arising from financial constraints (i.e., difficulties paying bills), poor housing quality (i.e., overcrowding), deprived neighborhood conditions (i.e., noise, feeling unsafe), and poor working conditions (i.e., job insecurity) [10]. Those with a lower SEP also more frequently experience psychosocial hassles resulting from interpersonal contact, such as being stigmatized or discriminated against [10].

The experience of daily hassles has been associated with poorer physical health outcomes [8, 12] and with unhealthy food consumption [13, 14]. Daily hassles may influence health via biological processes (e.g., elevated stress hormones) and cognitive processes. Previous research on the cognitive influence of stressors on socioeconomic inequalities in health has focused on inequalities in e.g., coping skills, control over health, and health motivation [15, 16]. Motivation has been studied in terms of preferences, attitudes and expectations for health and longevity [17]. Whether dealing with severe and urgent daily hassles influences people’s motivation for health, and more specifically, whether people who experience more daily hassles would consciously consider goals such as striving for a long life and staying healthy as less pressing and less focal, has not yet been studied. A lower perceived importance of health could affect health-promoting behaviors and, eventually, one’s health. It could also explain why those with a lower SEP may have a lower uptake of, and high drop-out from, health-related interventions [18]. In line with the work of Wiggins [19], this hypothesis was formed by linking the scarcity theory with the goal conflict theory.

The scarcity theory explains that the experience of having scarce resources, which is more common among people with a lower SEP, directs people’s attention away from long-term goals toward managing the scarcity and associated hassles [19–21]. The presence of daily hassles may hence reduce people’s mental bandwidth to focus and spend time on less pressing goals, especially when limited resources are available [22]. The goal conflict theory suggests that, when faced with scarce resources and daily hassles, the presence of goal conflicts is also likely to increase [23, 24]. Someone who experiences daily hassles (e.g., pressing work, household, or financial hassles) may find that goals related to resolving these hassles conflict with health-related goals, which are often considered more abstract and less urgent [25]. As a consequence of the experience of scarcity and goal conflicts, taking care of one’s health to prevent diseases or to increase one’s healthy life span could become less focal [5, 19, 26], which may lead to poorer health outcomes [27].

**Fig. 1:**
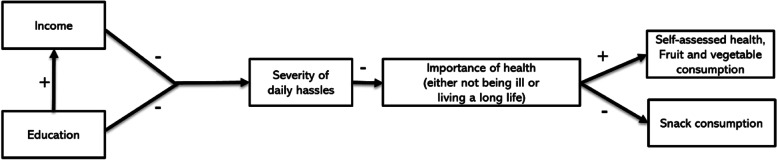
The conceptual sequential mediation model and anticipated directions of hypothesized associations tested in this study

This study cross-sectionally tests the hypothesis that a lower income or educational level is linked with the experience of more severe daily hassles, which is assumed to be sequentially associated with a lower perceived importance of not being ill or living a long life. These health goals, in turn, are expected to be associated with lower self-assessed health (SAH), lower fruit and vegetable consumption, and higher snack consumption (see Fig. 1 for our conceptual model). Since many public health policies and interventions target health outcomes and health behaviors directly, it is relevant to understand if this angle is considered important to engage in by people who experience double burdens (e.g., people with a lower SEP, who may frequently face structural challenges such as daily hassles). This exploratory, cross-sectional study could offer an initial understanding of the associations between SEP, daily hassles, and the perceived importance of health. This could stimulate further lines of research, and could contribute to policy recommendations that are in line with people’s needs and priorities.

## Methods

### Design and study population

Cross-sectional data were collected in the Netherlands via an online survey. An invitation to participate in the survey was sent to members of Flycatcher, an online research panel [28]. The survey was completed in two parts: the first half of the survey was completed in January 2019 (this included most variables used for the current study, except food consumption) and the second half was completed in February 2019 (including food consumption). Inclusion criteria were being between 25 and 60 years of age, and not currently being enrolled in full-time education. In total, 1,336 participants (59% response rate) consented and participated in the first part of the survey. The second part was completed by 1,051 of these 1,336 participants (79% response rate).

Data on food consumption for the 285 respondents who only participated in the first wave of the survey were imputed. Imputation has been suggested to provide nearly unbiased results for variables missing at random [29]. The multiple imputation was based on all variables used in our analyses, thus correcting the imputed outcomes for potential gender, age, SEP, and SAH differences between respondents and non-respondents, and 20 imputed datasets were used. Six participants were removed from the analyses because of missing values on educational level (*n* = 4) or gender (*n*= 2), resulting in an analytic sample of 1,330. The sample was representative of the Dutch population in gender, age, educational level, and province of residence [30]. Participants with low incomes were oversampled to compensate for their potential relatively lower response rate and were overrepresented in the final sample compared to the Dutch population [31]. Ethical approval for this study was obtained from the ethics committee of the Faculties of Science and Geo Sciences, Utrecht University (GEO FETC18-014).

### Measures

#### Socioeconomic position

Level of education and household equivalent income level were used as separate proxies for SEP, as both are commonly used indicators of SEP[7]. Following the International Standard Classification of Education (ISCED) 2011 [32], self-reported highest attained educational level was categorized as low (lower secondary education at most; ISCED 0–2), intermediate (upper secondary education at most; ISCED 3–4), or high (tertiary education or beyond; ISCED 5–6). All members of the online research panel register their gross household income with the online panel annually. To account for household composition and size, the research agency transformed this into household equivalent income by dividing household income by the number of members in a household. In this calculation, adults were assigned a higher weight than children because of their higher consumption rates. Household equivalent income level (further referred to as income level) was then categorized into low (< €13,300 per year – due to oversampling in the low income category, this group consists of extremely low incomes), middle (€13,300 to €41,200 – this group consists of low to modal incomes), and high (> €41,200 per year – this group consists of households with modal incomes and higher). The cut-off points were based on those developed by Statistics Netherlands [33].

#### The severity of daily hassles

The severity of experienced daily hassles was measured with the question “To what extent did the following hassles cause you concern or worries in the previous four weeks?” Based on two hassle scales [34, 35] and a daily hassles list developed specifically for people in lower SEPs in the United States [10], participants were presented with a summarized list of ten hassles that were deemed suitable for the Dutch context. These were: legal issues, household issues, issues being organized, issues with paperwork (paying bills, filling in forms), issues with the maintenance or state of your home, job stress issues, job security issues, noise pollution issues, personal safety issues, and issues with being contradicted or discriminated against. For each item, four response options were available: “not applicable/ no hassles”, “some hassles”, “quite some hassles”, and “many hassles”. Following Kanner et al. (1981), the severity of the ten daily hassles was calculated based on the mean of the four-point ratings (Cronbach’s α = 0.83) [34]. The resulting score ranged from 0 (= “no daily hassles”) to 4 (= ” many daily hassles”).

#### Perceived importance of health

A qualitative study in the Netherlands found that those with a lower level of education were more likely to conceptualize health in negative terms (e.g., “absence of disease”), while those with a higher education included more positive aspects (e.g., “lust for life”) [36]. Therefore, following the survey question “How important are the following things to you?”, we listed two distinct conceptualizations of health: “not being ill” (negative framing) and “living a long life” (positive framing). Responses were given on a five-point scale (from 1 = “very unimportant” to 5 = “very important”). The two items were included in the analysis as two separate indicators of the perceived importance of health.

#### Self-assessed health

SAH is considered a reliable indicator of health status and captures people’s ability to live a life without functional limitations [37]. SAH was measured by asking, “In general, how do you perceive your health?”. Participants answered on a five-point scale, with response options ranging from 1 (= “poor”) to 5 (= “excellent”).

#### Food consumption

Food consumption was measured using a food frequency questionnaire [38]. Two outcome measures were created: the total number of fruits and vegetables consumed (FVC) per week and the total number of snacks consumed per week since these two measures are likely driven by different underlying factors [39]. Participants reported how many days per week they usually consumed fruits and vegetables, and small and large, sweet and savory snacks with response options ranging from “never” to “seven days per week”. Additionally, participants were asked to indicate how many portion sizes they would have on a typical day they consumed that type of food, with response options ranging from “less than one a day” to “more than five a day”. Definitions of what constitutes one portion size were provided and were specific to the food product. For example, one apple qualified as one portion of fruit. The multiplication of frequency and quantity of portions allowed us to calculate a continuous measure of participants’ healthy and unhealthy food consumption per week. Regarding snack consumption, large and small snacks were summed into one overall variable for total snack consumption, with large snacks given twice the weight of small snacks. In our sample, FVC ranged from 0 to 84 portions per week, and snack consumption ranged from 0 to 180 portions per week. The snack consumption outcome was transformed by taking the square root to normalize its distribution. All analyses were run using transformed snack consumption data, and sensitivity analyses were run using untransformed snack consumption data.

#### Socio-demographic covariates

Age in years was included as a continuous variable. Gender (1 = “female”, 0 = “male”), being in paid employment (1 = “yes”, 0 = “no”), and living with a partner (1 = “yes”, 0 = “no”) were included as binary variables. Age, gender, and living with a partner were modeled to confound the SEP variables, the mediators and the outcomes. Being in paid employment was modelled to confound income level, the mediators, and the outcomes.

### Statistical analyses

Structural equation models (SEMs) were built to examine whether the severity of daily hassles and the perceived importance of health mediated the relationships between SEP and SAH, SEP and FVC, and SEP and snack consumption, both individually and sequentially. Separate models were constructed for each outcome variable. A pathway between the two SEP indicators was included in all models, as educational level likely influences household equivalent income level [40]. (These variables were indeed correlated in our data, see Additional file 1.) Potential confounding effects of age, gender, paid employment, and living with a partner were taken into account in the SEMs, as previous research points to their influence on food consumption [41] and the perceived importance of health [36, 42] and because they correlated with SEP (see Additional file 1).

A step-by-step procedure was used to test for mediation [43]. First, a baseline model was created to identify if there was a relationship between the dependent and independent variables in the presence of covariates. Second, models including each of the mediators individually were analyzed. Third, a sequential mediation relationship was tested. In addition to the main models that used a combined severity of daily hassles variable, supplementary mediation models were run for each specific daily hassle with the outcome variables.

All outcome variables were treated as continuous, so all SEMs were fitted with the Maximum Likelihood estimator. While SAH was categorical, it was treated as continuous because of its normal distribution over five categories [44]. Treating SAH as categorical or using a robust Maximum Likelihood estimator did not impact our model estimates. SAH mediation parameters were bootstrapped 10,000 times. The decision tree by Zhao et al. (2010) was used to establish if there was mediation [43]. Also, their classifications of complementary mediation (indirect and direct effects in the same direction), competitive mediation (indirect and direct effects in opposite directions), and indirect-only mediation (indirect, but no direct, effects) were used to classify the type of mediation [43]. All analyses were run in Mplus version 8.4 [45].

## Results

### Participant characteristics

Table 1 shows the descriptive statistics of the sample. Most participants had a middle educational level and a low-income level. Also, most participants were in paid employment, and just over half of the participants lived with a partner. Slightly more women participated in the study, and the average age was 45 years. Correlations between variables can be found in Additional file 1.

**Table 1:** Descriptive statistics of the study sample (based on imputed data)

	**Percentage per category or** **mean, standard deviation (range)**
**Educational level**	
Low	26.9%
Middle	45.8%
High	27.3%
**Income level**	
Low	39.5%
Middle	30.4%
High	30.2%
**Female**	57.4%
**In paid employment**	62.7%
**Living with a partner**	54.3%
**Participant age**	44.9, 10.4 (25–60)
**Severity of daily hassles**	1.6, 0.5 (1–4)
**Importance of not being ill**	
Very unimportant	0.4%
Unimportant	0.7%
Neutral	4.6%
Important	43.2%
Very important	51.1%
**Importance of a long life**	
Very unimportant	2.0%
Unimportant	6.3%
Neutral	24.1%
Important	51.5%
Very important	16.1%
**Self-assessed health**	
Very poor	6.0%
Poor	27.0%
Neutral	45.5%
Good	16.8%
Excellent	4.7%
**Fruit and vegetable consumption (portions per week)**	24.8, 13.3 (0–84)
**Snack consumption (square root of portions per week)**	4.1, 2.0 (0–180)

### Mediation analyses

All estimates of the single mediation analyses are shown in Additional file 2, all estimates of the sequential mediation analyses are shown in Additional file 3, and all mediation effects are shown in Additional file 4. Note that our models had conflicting model fit statistics; this was likely due to the complexity of the models combined with low degrees of freedom [46].

#### Mediation results for self-assessed health

The baseline model (Additional file 2) showed a small positive association between educational level and SAH, while income level did not have a significant association with SAH despite having a similar estimate. In the next step, the severity of daily hassles was assessed as a potential single mediator of the association between SEP and SAH. The severity of daily hassles did not mediate the pathway between educational level and SAH, as educational level was not associated with the severity of daily hassles (see Additional file 2). Income level was negatively associated with the severity of daily hassles, and the severity of daily hassles was negatively associated with SAH. Given the lack of a direct effect from income level to SAH in our data (direct effect: 0.02 *p* > 0.10), indirect-only mediation between income level and SAH via the severity of daily hassles was identified (indirect effect: 0.04, *p* < 0.01, total effect: 0.06, *p* < 0.10, see Additional file 4). Mediation models for specific daily hassles showed that each daily hassle separately mediated the relationship between income level and SAH. The largest indirect-only mediation effect size was found for paperwork-related hassles (see Additional file 5).

The perceived importance of not being ill was assessed as a potential single mediating variable in model 2B (Additional file 2). Educational level had a small positive association with the perceived importance of not being ill, which, in turn, was positively associated with SAH (indirect effect: 0.01, *p* < 0.01, direct effect: 0.06, *p* < 0.10, total effect: 0.07, *p* < 0.05, see Additional file 4). These findings imply that there was complementary mediation between educational level and SAH via the perceived importance of not being ill. Income level was not associated with the perceived importance of not being ill.

The perceived importance of living a long life was tested as a potential single mediating variable in model 2C (Additional file 2). Educational level had a small positive association with the perceived importance of living a long life, which, in turn, was negatively associated with SAH (indirect effect: -0.01, *p* < 0.01, direct effect: 0.07, *p* < 0.05, total effect: 0.07, *p* < 0.05, see Additional file 4). The opposite directions of the effects imply competitive mediation of the perceived importance of living a long life on the relationship between educational level and SAH. Income level was not associated with the perceived importance of living a long life.

**Fig. 2:**
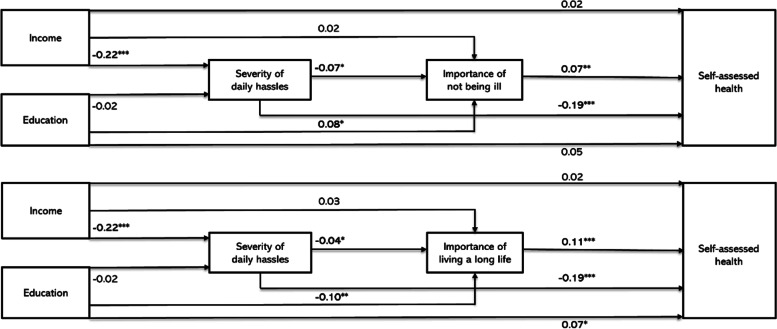
Overview of the main path estimates for the sequential mediation model between socioeconomic position and self-assessed health. Presented are SEM estimates *P < 0.05, ** *P* < 0.01, *** *P* < 0.001

Sequential mediation of the association between SEP and SAH through the severity of daily hassles and, subsequently, the perceived importance of health were tested in models 3A and 3B (Fig. 2, and Additional file 3). Although the SEM estimates indicated a potential pathway from income to SAH through the severity of daily hassles followed by the perceived importance of not being ill, the estimated mediation effects were null and insignificant (indirect effect of 0.00, *p* > 0.10, direct effect of 0.02, *p* > 0.10, and total effect of 0.06 *p* < 0.10, see Additional file 4). No sequential mediation was identified between the severity of daily hassles and the perceived importance of living a long life.

#### Mediation analyses of fruit and vegetable consumption

The baseline model (Additional file 2) showed that educational and income level were each positively associated with FVC. In the next step, the severity of daily hassles did not mediate the relationship between educational level and FVC, since educational level was not associated with the severity of daily hassles (see Additional file 2). The severity of daily hassles had a small negative association with FVC. The severity of daily hassles partly mediated the relationship between income level and FVC ( indirect effect: 0.02, *p* < 0.05, total effect: 0.09, *p* < 0.05, see Additional file 4). The insertion of daily hassles into the model had a small negative influence on the direct estimate of the relationship between income and FVC. As a result, income level was no longer statistically significantly associated with FVC (direct effect: 0.07, *p* > 0.10, see Additional file 4), indicating weak evidence for indirect-only mediation. Mediation models for specific daily hassles showed only indirect-only mediation via household, paperwork, and personal safety hassles, none of the other specific hassles mediated the relationship between income level and FVC (see Additional file 5).

**Fig. 3:**
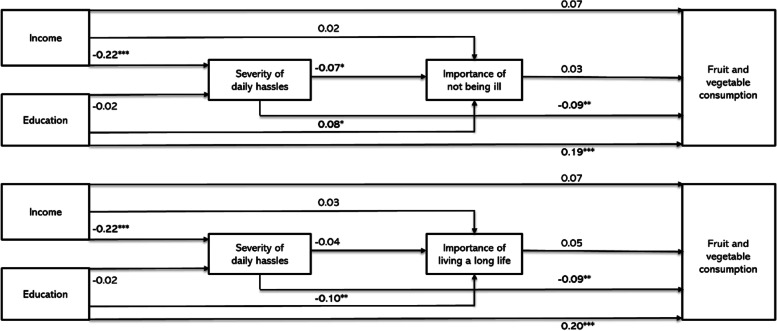
Overview of the main path estimates for the sequential mediation model between socioeconomic position and fruit and vegetable consumption. Presented are SEM estimates *P < 0.05, ** *P* < 0.01, *** *P* < 0.001

The perceived importance of not being ill and living a long life were assessed as potential single mediating variables in models 2B and 2C (Additional file 2). However, neither one of the perceived importance of health measures was associated with FVC. No single or sequential mediation effects (see Fig. 3, and Additional file 3 and 4) were found.

#### Mediation results for snack consumption

**Fig. 4:**
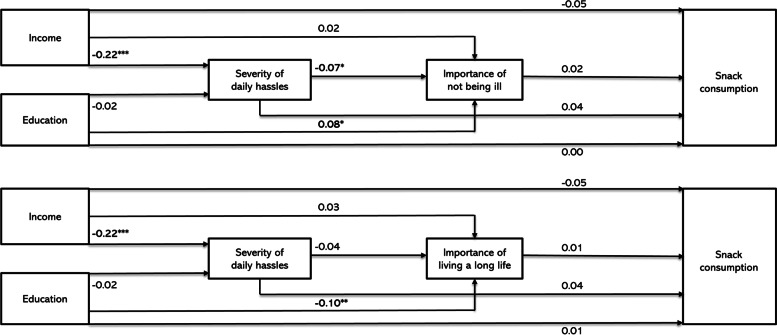
Overview of the main path estimates for the sequential mediation model between socioeconomic position and snack consumption. Presented are SEM estimates *P < 0.05, ** *P* < 0.01, *** *P* < 0.001

The baseline model with confounders showed that neither educational level nor income level were associated with snack consumption (see Additional file 2). Also, none of the mediators were associated with snack consumption, and no evidence of mediation was found (see Fig. 4, and Additional file 3 and 4). To assess the sensitivity of analyses to variable coding, these analyses were also run using the untransformed snack consumption variable, with similar results (see Additional file 7).

## Discussion

This study explored if a lower income or educational level was linked with the experience of more severe daily hassles, and if this would sequentially be associated with a lower perceived importance of not being ill or living a long life, and if, in turn, this would be associated with lower self-assessed health (SAH), lower fruit and vegetable consumption, and higher snack consumption. We identified educational inequalities in SAH and FVC, but not in snack consumption. Income inequalities were identified in FVC, but not in SAH and snack consumption. Based on this cross-sectional study, we found no evidence for sequential mediation through the severity of daily hassles *and* the perceived importance of health for the observed educational and income inequalities. However, our findings show that the severity of daily hassles may mediate income inequalities in SAH and FVC, and that the perceived importance of not being ill and of living a long life may mediate educational inequalities in SAH.

Before interpreting our findings, several limitations should be taken into account. This cross-sectional study provides preliminary, explorative insights into potential pathways explaining health inequalities. Although the temporal relationships assessed in this study can be deemed theoretically plausible, no definitive conclusion regarding the rejection of our null hypothesis can be drawn because of our cross-sectional design. Moreover, endogeneity, and more specifically, simultaneity bias is likely present in our model since (a) educational level may influence the confounders included in the model, especially one’s employment status, which in turn could influence the household income level, and (b) a lower SAH could also lead to a lower perceived importance of health, more daily hassles, and a lower SEP. This potential endogeneity is further inflated by potentially omitted variables, such as other factors related to health decision-making, or other factors hidden to the researcher. Although our SEM reduced some of the estimation biases by fitting all equations simultaneously [47], the estimates for the severity of daily hassles, the perceived importance of health, and our confounders, may be overestimated due to endogeneity issues.

Likewise, given the lack of a validated measure for the perceived importance of health, interpretational issues by respondents regarding the meaning of not being ill and living a long life cannot be ruled out. Moreover, its interpretation may also systematically differ between socioeconomic groups [36, 48]. Furthermore, although we found no evidence of an association between the severity of daily hassles and an (cognitively salient) appraisal of the importance of health, it is still possible that this relationship is partly unconsciously; people may instinctively place more emphasis on their most salient or pressing needs, while still (consciously) placing a high value on health. In a previous study using the same sample [49], we analyzed the relative perceived importance of health compared to other life domains, such as financial situation. We found that people with a lower socioeconomic position were somewhat more likely to give a higher importance to their financial situation than to their health. However, it remains unclear if a lower conscious perceived importance of health translates into different (subconscious) trade-offs or priorities in daily life. Finally, the use of self-reports regarding daily hassles can be problematic, especially in the case of socioeconomic differences in severity perceptions. It has been suggested that people adapt their stress tolerance and perception to the circumstances they are in [50]. If this is the case, the socioeconomic inequalities in the severity of daily hassles we identified may be underestimated. Given these limitations, we are cautious to definitively reject the null hypothesis that the severe experience of daily hassles does not lead to health consciously being perceived as less important.

Despite these limitations, our results clearly indicate that people with lower incomes experienced more severe daily hassles than those with higher incomes, while a lower educational level was not associated with increased severity of daily hassles. An explanation for the potential relationship between income, and not education, with the severity of hassles, could be that living on a lower income may be direct barrier to obtaining services to deal with or reduce daily hassles. E.g., those on a lower income are less likely to be able to afford a cleaner, or a builder to upgrade living conditions, and therefore cannot easily reduce hassles associated with household issues (e.g., unclean house, poor maintenance).

The lack of a direct effect between income level and SAH or FVC after daily hassles were included in the model, highlights how important daily hassles could be in relation to health outcomes and behavior. Although the consumption of high-fat foods has been linked with daily hassles in other studies [51, 52], the severity of daily hassles was not associated with snack consumption in our study. This may indicate that FVC is more easily disrupted by the severity of daily hassles than snack consumption. FVC generally requires more effort (e.g., peel, slice, or cook) than consuming ready-to-eat snacks. Therefore, the experience of severe daily hassles possibly inhibits the active choice of FVC more than it increases snacking. The mediating effects of the severity of daily hassles show that to prevent growing income inequalities in health and healthy food consumption, it is important to address the structural conditions, associated with a lower SEP, that may cause daily hassles [53].

Our results indicated that groups with a higher education considered not being ill *more* important than groups with a lower education, yet, groups with a higher education considered living a long life *less *important than groups with a lower education. It could be that people who e.g., more often encounter premature deaths in their surroundings, which is more common among those with a lower SEP (given observed inequalities in life expectancy) [3], may internalize this into considering a long life more important. This finding may also reflect a form of privilege, as those with a higher educational level may anticipate living long lives.

Instead of the sequential mediation pathway we hypothesized, a moderated mediation pathway could have been an alternative hypothesis. According to the goal conflict theory, the association between the perceived importance of health and health outcomes could also depend on, or be undermined by, the severity of daily hassles [22]. In this scenario, someone could consider health important yet, due to being consumed by severe daily hassles, be unable to translate this into healthy food consumption or other health-promoting actions, resulting in poorer health. A potential moderated mediation pathway was therefore tested in additional analyses (see Additional file 6). We found no evidence of an interaction between the severity of daily hassles and the perceived importance of health in their effects on SAH, FVC, and snack consumption.

Future studies on this topic should ideally use an experimental design to study the (unconscious) trade-off between health and other pressing issues in people’s daily lives. Alternatively, future studies could include an instrumental variable to address endogeneity. The instrumental variable should directly influence SEP yet be unrelated to the health outcomes. These variables were not present in our data set. The goal conflict model and the resource capacity model suggest that other factors not incorporated in this study, such as the desirability of a goal and the amount of effort required to pursue a goal [22], or control perceptions and social support, may also have played a role in the relationship between the severity of daily hassles and the perceived importance of health [23, 54]. These factors might be of interest to include in future studies. To resolve these interpretational issues, future research should qualitatively assess how different socioeconomic groups conceptualize health and its role and importance in the trade-offs people make in their daily lives. Since the sample used was representative of the Dutch adult population, our results are generalizable to the Dutch context, and may be generalizable to countries who share similarities in terms of their educational and welfare systems, which influence people’s SEP, may lead to similar daily hassles and administrative burdens, and may consequently have a similar influences on FVC and SAH. Our final recommendation is to study this hypothesis across different contexts.

## Conclusions

This explorative study identified educational inequalities in SAH, and income and educational inequalities in FVC. We found no evidence of sequential mediation through the severity of daily hassles *and* the perceived importance of health for these educational and income inequalities. However, the severity of daily hassles mediated income inequalities in SAH and FVC, and the perceived importance of health mediated educational inequalities in SAH. This suggests that the unfavorable circumstances people with a lower SEP are structurally exposed to may lead to poorer health in various ways, but having to deal with these unfavorable circumstances does not lead to health consciously being perceived as less important. To protect and improve the health of populations with a lower SEP, we recommend that basic needs, such as e.g., safe and healthy housing, and sufficient financial means, be provided, and paperwork hassles need to be reduced before these needs turn into hassles. Reducing these daily hassles can benefit health and health behaviors, especially for those with a lower SEP.

## Supplementary Information

**Additional file 1.**

**Additional file 2.**

**Additional file 3.**

**Additional file 4.**

**Additional file 5.**

**Additional file 6.**

**Additional file 7.**

## Data Availability

The datasets used and/or analyzed during the current study are available from the corresponding author upon reasonable request.

## Abbreviations

FVC: Fruit and vegetable consumption
SAH: Self-assessed health
SEP: Socioeconomic position
