# Supplementary material for: Socioeconomic inequalities in self-assessed health and food consumption: the mediating roles of daily hassles and the perceived importance of health
Source: BMC Public Health. 2023 Mar 7;23:439. doi: 10.1186/s12889-023-15077-0 (PMC9990278; doi:10.1186/s12889-023-15077-0)
Supplement: Supplementary file 1 — Additional file 1. [file 12889_2023_15077_MOESM1_ESM.docx]

**Additional file 1: Bivariate correlations of variables**

| Variable | 1 | 2 | 3 | 4 | 5 | 6 | 7 | 8 | 9 | 10 | 11 | 12 |
| --- | --- | --- | --- | --- | --- | --- | --- | --- | --- | --- | --- | --- |
| 1. Educational level | 1 |  |  |  |  |  |  |  |  |  |  |  |
| 2. Income level | .30^a^* | 1 |  |  |  |  |  |  |  |  |  |  |
| 3. Female | -.07^c^* | -.30^c^* | 1 |  |  |  |  |  |  |  |  |  |
| 4. In paid employment | .31^c^* | .50^c^* | -.19^b^* | 1 |  |  |  |  |  |  |  |  |
| 5. Living with a partner | .14^c^* | .60^c^* | -.17^b^* | .34^b^* | 1 |  |  |  |  |  |  |  |
| 6. Age | -.28^a^* | -.06^a^* | -.14^d^* | -.18^d^* | -.00^d^ | 1 |  |  |  |  |  |  |
| 7. Severity of daily hassles | 0.00^a^ | -.18^a^* | .08^c^* | -.08^c^* | -.19^c^* | -.09^a^* | 1 |  |  |  |  |  |
| 8. Importance of not being ill | .04^a^ | .04^a^ | -.00^c^ | .03^c^ | .03^c^ | .05^a^* | -.05*^a^ | 1 |  |  |  |  |
| 9. Importance of a long life | -.05^a^* | .09^a^* | -.08^c^* | .10^c^* | .13^c^* | .04^a^ | -.07^a^* | .27^a^* | 1 |  |  |  |
| 10. Self-assessed health | .22^a^* | .29^a^* | -.12^c^* | .44^c^* | .26^c^* | -.17^a^* | -.19^a^* | .08^a^* | .13^a^* | 1 |  |  |
| 11. Fruit and vegetable consumption | .17^f^* | .12^f^* | .01^e^ | .07^e*^ | .06^e*^ | 0.04^f^ | -.13^f^* | .07^f*^ | .05^f^ | .17^f^* | 1 |  |
| 12. Snack consumption | 0.02^f^ | -.01^f^ | -.02^e^ | .05^e^ | 0.01^e^ | -.03^f^ | .04^f^ | .01^f^ | .01^f^ | -.05^f^ | -.09^f^* | 1 |
