# Supplementary material for: Socioeconomic inequalities in self-assessed health and food consumption: the mediating roles of daily hassles and the perceived importance of health
Source: BMC Public Health. 2023 Mar 7;23:439. doi: 10.1186/s12889-023-15077-0 (PMC9990278; doi:10.1186/s12889-023-15077-0)
Supplement: Supplementary file 2 — Additional file 2. [file 12889_2023_15077_MOESM2_ESM.docx]

**Additional file 2: Standardized coefficients of structural equation models.**

Table 1: Standardized coefficients of structural equation models for the outcome self-assessed health

| Model: | Outcome: | Predictor: | Estimates (Standard Error): |
| --- | --- | --- | --- |
| Model 1= Baseline  Chi-square (degrees of freedom=1)=65.23, p=0.00  Comparative Fit Index=0.96 Tucker-Lewis Index=0.39 Root Mean Square Error of Approximation=0.22 Standardized Root Mean Square Residual=0.04 | Self-assessed health | Income level | 0.06(0.04) |
|  |  | Educational level | 0.06(0.03)* |
|  |  | In paid employment | 0.31(0.03)*** |
|  |  | Age | -0.14(0.03)*** |
|  |  | Female | -0.04(0.03) |
|  |  | Living with a partner | 0.10(0.03)** |
| Model 2A=Model 1 + Mediator Daily hassles  Chi-square (degrees of freedom=1)=65.23, p=0.00 Comparative Fit Index=0.96 Tucker-Lewis Index=0.20 Root Mean Square Error of Approximation=0.22  Standardized Root Mean Square Residual=0.04 | Self-assessed health | Income level | 0.02(0.04) |
|  |  | Educational level | 0.06(0.03) |
|  |  | In paid employment | 0.31(0.03)*** |
|  |  | Age | -0.16(0.03)*** |
|  |  | Female | -0.04(0.03) |
|  |  | Living with a partner | 0.09(0.03)** |
|  |  | Severity of daily hassles | -0.19(0.03)*** |
|  | Severity of daily | Income level | -0.22(0.03)*** |
|  | hassles | Educational level | -0.02(0.03) |
|  |  | In paid employment | 0.01(0.03) |
|  |  | Participant age | -0.13(0.03)*** |
|  |  | Female | -0.04(0.03) |
|  |  | Living with a partner | -0.07(0.03)* |
| Model 2B=Model 1 + Mediator not being ill  Chi-square (degrees of freedom=1)=65.23, p=0.00 Comparative Fit Index=0.96 Tucker-Lewis Index=0.11  Root Mean Square Error of Approximation=0.22  Standardized Root Mean Square Residual=0.04 | Self-assessed | Income level | 0.06(0.04) |
|  | health | Educational level | 0.06(0.03) |
|  |  | In paid employment | 0.31(0.03)*** |
|  |  | Participant age | -0.14(0.03)*** |
|  |  | Female | -0.04(0.03) |
|  |  | Living with a partner | 0.10(0.03)** |
|  |  | Not being ill | 0.08(0.02)** |
|  | Not being ill | Income level | 0.03(0.04) |
|  |  | Educational level | 0.08(0.03)** |
|  |  | In paid employment | 0.02(0.04) |
|  |  | Participant age | 0.09(0.03)** |
|  |  | Female | 0.02(0.03) |
|  |  | Living with a partner | 0.01(0.04) |
| Model 2C=model 1 + Mediator Living a long life  Chi-square (degrees of freedom=1)=65.23, p=0.00 Comparative Fit Index=0.96 Tucker-Lewis Index=0.13  Root Mean Square Error of Approximation=0.22  Standardized Root Mean Square Residual=0.04 | Self-assessed health | Income level | 0.06(0.04) |
|  |  | Educational level | 0.07(0.03)* |
|  |  | In paid employment | 0.30(0.03)*** |
|  |  | Participant age | -0.14(0.03)*** |
|  |  | Female | -0.04(0.03) |
|  |  | Living with a partner | 0.09(0.03)** |
|  |  | A long life | 0.12(0.03)*** |
|  | A long life | Income level | 0.04(0.04) |
|  |  | Educational level | -0.10(0.03)** |
|  |  | In paid employment | 0.10(0.03)** |
|  |  | Participant age | 0.03(0.03) |
|  |  | Female | -0.02(0.03) |
|  |  | Living with a partner | 0.08(0.03)* |

* p<0.05, ** p<0.01, *** p < 0.001.

Table 2: Standardized coefficients of structural equation models for the outcome Fruit and vegetable consumption.

| Model: | Outcome: | Predictor: | Imputed estimates (Standard Error): |
| --- | --- | --- | --- |
| Model 1= Baseline  Chi-square (degrees of freedom=1)=65.38, p=0.00  Comparative Fit Index=0.94 Tucker-Lewis Index=0.13 Root Mean Square Error of Approximation=0.22 Standardized Root Mean Square Residual=0.04 | Fruit and | Income level | 0.09(0.04)* |
|  | vegetable | Educational level | 0.20(0.04)*** |
|  | consumption | In paid employment | -0.00(0.04) |
|  |  | Participant age | 0.13(0.04)*** |
|  |  | Female | 0.07(0.03)* |
|  |  | Living with a partner | -0.00(0.04) |
| Model 2A=Model 1 + Mediator Daily hassles  Chi-square (degrees of freedom=1)=65.45, p=0.00 Comparative Fit Index=0.95 Tucker-Lewis Index=0.00 Root Mean Square Error of Approximation=0.22  Standardized Root Mean Square Residual=0.04 | Fruit and | Income level | 0.07(0.04) |
|  | vegetable | Educational level | 0.20(0.04)*** |
|  | consumption | In paid employment | 0.00(0.04) |
|  |  | Participant age | 0.11(0.04)** |
|  |  | Female | 0.07(0.03)* |
|  |  | Living with a partner | -0.01(0.04) |
|  |  | Severity of daily hassles | -0.09(0.03)* |
|  | Severity of daily | Income level | -0.22(0.04)*** |
|  | hassles | Educational level | -0.02(0.03) |
|  |  | In paid employment | 0.01(0.03) |
|  |  | Participant age | -0.13(0.03)*** |
|  |  | Female | -0.04(0.03) |
|  |  | Living with a partner | -0.07(0.03)* |
| Model 2B=Model 1 + Mediator not being ill  Chi-square (degrees of freedom=1)=65.42, p=0.00 Comparative Fit Index=0.95 Tucker-Lewis Index=0.00 Root Mean Square Error of Approximation=0.22  Standardized Root Mean Square Residual=0.04 | Fruit and | Income level | 0.09(0.04)* |
|  | vegetable | Educational level | 0.19(0.04)*** |
|  | consumption | In paid employment | -0.00(0.04) |
|  |  | Participant age | 0.12(0.04)** |
|  |  | Female | 0.07 (0.03)* |
|  |  | Living with a partner | -0.00(0.04) |
|  |  | Not being ill | 0.04(0.03) |
|  | Not being ill | Income level | 0.03(0.04) |
|  |  | Educational level | 0.08(0.03)** |
|  |  | In paid employment | 0.02(0.03) |
|  |  | Participant age | 0.09(0.03)** |
|  |  | Female | 0.02(0.03) |
|  |  | Living with a partner | 0.01(0.03) |
| Model 2C=model 1 + Mediator Living a long life  Chi-square (degrees of freedom=1)=65.42, p=0.00 Comparative Fit Index=0.95 Tucker-Lewis Index=0.00  Root Mean Square Error of Approximation=0.22  Standardized Root Mean Square Residual=0.04 | Fruit and | Income level | 0.09(0.04)* |
|  | vegetable | Educational level | 0.20(0.04)*** |
|  | consumption | In paid employment | 0.01 (0.04) |
|  |  | Participant age | 0.12(0.04)*** |
|  |  | Female | 0.07(0.03)* |
|  |  | Living with a partner | -0.01(0.04) |
|  |  | A long life | 0.06(0.03) |
|  | A long life | Income level | 0.04(0.04) |
|  |  | Educational level | -0.10(0.03)** |
|  |  | In paid employment | 0.10(0.03)** |
|  |  | Participant age | 0.03(0.03) |
|  |  | Female | -0.02(0.03) |
|  |  | Living with a partner | 0.08(0.03)* |

* p<0.05, ** p<0.01, *** p < 0.001.

Table 3: Standardized coefficients of structural equation model with transformed outcome snack consumption.

| Model: | Outcome: | Predictors: | Imputed estimates (Standard Error): |
| --- | --- | --- | --- |
| Model 1= Baseline  Chi-square (degrees of freedom=1)=65.31, p=0.00  Comparative Fit Index=0.94 Tucker-Lewis Index=0.13 Root Mean Square Error of Approximation=0.22 Standardized Root Mean Square Residual=0.04 | Snack consumption | Income level | -0.06(0.05) |
|  |  | Educational level | 0.00(0.04) |
|  |  | In paid employment | 0.07(0.04) |
|  |  | Participant age | -0.02(0.03) |
|  |  | Female | -0.02(0.03) |
|  |  | Living with a partner | 0.01(0.04) |
| Model 2A=Model 1 + Mediator Severity of daily hassles  Chi-square (degrees of freedom=1)=65.35, p=0.00 Comparative Fit Index=0.95  Tucker-Lewis Index=0.00 Root Mean Square Error of Approximation=0.22 | Snack consumption | Income level | -0.05(0.05) |
|  |  | Educational level | 0.00(0.04) |
|  |  | In paid employment | 0.07(0.04) |
|  |  | Participant age | -0.01(0.03) |
|  |  | Female | -0.02(0.03) |
|  |  | Living with a partner | 0.02(0.04) |
| Standardized Root Mean Square Residual=0.04 |  | Severity of daily hassles | 0.04(0.03) |
|  | Severity of daily hassles | Income level | -0.22(0.04)*** |
|  |  | Educational level | -0.02(0.03) |
|  |  | In paid employment | 0.01(0.03) |
|  |  | Participant age | -0.13(0.03)*** |
|  |  | Female | -0.04(0.03) |
|  |  | Living with a partner | -0.07(0.03)* |
| Model 2B=Model 1 + Mediator not being ill  Chi-square (degrees of freedom=1)=65.33, p=0.00  Comparative Fit Index=0.94 Tucker-Lewis Index=0.00 Root Mean Square Error of Approximation=0.22  Standardized Root Mean Square Residual=0.04 | Snack consumption | Income level | -0.06(0.05) |
|  |  | Educational level | 0.00(0.04) |
|  |  | In paid employment | 0.07(0.04) |
|  |  | Participant age | -0.02(0.03) |
|  |  | Female | -0.02(0.03) |
|  |  | Living with a partner | 0.01(0.04) |
|  |  | Not being ill | 0.01(0.03) |
|  | Not being ill | Income level | 0.03(0.04) |
|  |  | Educational level | 0.08(0.03)** |
|  |  | In paid employment | 0.02(0.03) |
|  |  | Participant age | 0.09(0.03)** |
|  |  | Female | 0.02(0.03) |
|  |  | Living with a partner | 0.01(0.03) |
| Model 2C=model 1 + Mediator Living a long life  Chi-square (degrees of freedom=1)=65.34, p=0.00,  Comparative Fit Index=0.95 Tucker-Lewis Index=0.00 Root Mean Square Error of Approximation=0.22  Standardized Root Mean Square Residual=0.04 | Snack consumption | Income level | -0.06(0.05) |
|  |  | Educational level | -0.00(0.04) |
|  |  | In paid employment | 0.07(0.04) |
|  |  | Participant age | -0.02(0.03) |
|  |  | Female | -0.02(0.03) |
|  |  | Living with a partner | 0.01(0.04) |
|  |  | A long life | 0.01(0.03) |
|  | A long life | Income level | 0.04(0.04) |
|  |  | Educational level | -0.10(0.03)** |
|  |  | In paid employment | 0.10(0.03)** |
|  |  | Participant age | 0.03(0.03) |
|  |  | Female | -0.02(0.03) |
|  |  | Living with a partner | 0.08(0.03)* |

* p<0.05, ** p<0.01, *** p < 0.001.
