# Supplementary material for: Socioeconomic inequalities in self-assessed health and food consumption: the mediating roles of daily hassles and the perceived importance of health
Source: BMC Public Health. 2023 Mar 7;23:439. doi: 10.1186/s12889-023-15077-0 (PMC9990278; doi:10.1186/s12889-023-15077-0)
Supplement: Supplementary file 3 — Additional file 3. [file 12889_2023_15077_MOESM3_ESM.docx]

**Additional file 3: Sequential mediation SEM coefficients.**

The sequential mediation models presented in this Additional file are expansions based on the models presented in Additional file 2.

Table 1: Sequential mediation SEM coefficients for the outcome Self-assessed health

**Outcome Self-assessed health**

| Model: | Outcome: | Predictors: | Estimates (Standard Error): |
| --- | --- | --- | --- |
| Model 3a: Sequential mediation:  Hassles > not being ill  Chi-square (degrees of freedom=1)=65.23, p=0.00 Comparative Fit Index=0.96 Tucker-Lewis Index=0.00 Root Mean Square Error of Approximation=0.22 Standardized Root Mean Square Residual=0.03 | Self-assessed health | Income level | 0.02(0.04) |
|  |  | Educational level | 0.05(0.03) |
|  |  | Paid employment | 0.31(0.03)*** |
|  |  | Participant age | -0.17(0.03)*** |
|  |  | Female | -0.05(0.03) |
|  |  | Living with a partner | 0.09(0.03)** |
|  |  | Severity of daily hassles | -0.19(0.03)*** |
|  |  | Not being ill | 0.07(0.02)** |
|  | Severity of daily | Income level | -0.22(0.03)*** |
|  | hassles | Educational level | -0.02(0.03) |
|  |  | Paid employment | -0.01(0.03) |
|  |  | Participant age | -0.13(0.03)*** |
|  |  | Female | -0.04(0.03) |
|  |  | Living with a partner | -0.07(0.03)* |
|  | Not being ill | Income level | 0.02 (0.04) |
|  |  | Educational level | 0.08 (0.03)* |
|  |  | Paid employment | 0.02 (0.04) |
|  |  | Participant age | 0.08(0.03)* |
|  |  | Female | 0.02(0.03) |
|  |  | Living with a partner | -0.00(0.04) |
|  |  | Severity of daily hassles | -0.07(0.03)* |
| Model 3b: Sequential mediation:  Hassles > a long life  Chi-square (degrees of freedom=1)=65.23, p=0.00  Comparative Fit Index=0.96  Tucker-Lewis Index=0.00  Root Mean Square Error of Approximation=0.22  Standardized Root Mean Square Residual=0.03 | **Self-assessed health** | Income level | 0.02(0.04) |
|  |  | Educational level | 0.07(0.03)* |
|  |  | Paid employment | 0.30(0.03)*** |
|  |  | Participant age | -0.16(0.03)*** |
|  |  | Female | -0.04(0.03) |
|  |  | Living with a partner | 0.08(0.03)* |
|  |  | Severity of daily hassles | -0.19(0.03)*** |
|  |  | A long life | 0.11(0.03)*** |
|  | Severity of daily hassles | Income level | -0.22 (0.03)*** |
|  |  | Educational level | -0.02 (0.03) |
|  |  | Paid employment | 0.01 (0.03) |
|  |  | Participant age | -0.13(0.03)*** |
|  |  | Female | -0.04(0.03) |
|  |  | Living with a partner | -0.07(0.03)* |
|  | A long life | Income level | 0.03(0.04) |
|  |  | Educational level | -0.10(0.03)** |
|  |  | Paid employment | 0.10(0.04)** |
|  |  | Participant age | 0.02(0.03) |
|  |  | Female | -0.02(0.03) |
|  |  | Living with a partner | 0.08(0.03)* |
|  |  | Severity of daily hassles | -0.04(0.03) |

* p<0.05, ** p<0.01, *** p < 0.001.

Table 2: Sequential mediation SEM coefficients for the outcome Fruit and vegetable consumption

| Model: | Outcome: | Predictors: | Imputed estimates (Standard Error): |
| --- | --- | --- | --- |
| Model 3a: Sequential mediation:  Severity of daily hassles > not being ill Chi-square (degrees of freedom=1)=65.48, P=0.00 Comparative Fit Index =0.95 Tucker-Lewis Index=0.00 Root Mean Square Error of Approximation=0.22 Standardized Root Mean Square Residual=0.03 | Fruit and vegetable consumption | Income level | 0.07(0.04) |
|  |  | Educational level | 0.19(0.04)*** |
|  |  | Paid employment | 0.00(0.04) |
|  |  | Participant age | 0.11(0.04)** |
|  |  | Female | 0.07(0.03)* |
|  |  | Living with a partner | -0.01(0.04) |
|  |  | Severity of daily hassles | -0.09 (0.03)** |
|  |  | Not being ill | 0.03(0.03) |
|  | Severity of daily | Income level | -0.22(0.04)*** |
|  | hassles | Education | -0.02(0.03) |
|  |  | Employment | -0.01(0.03) |
|  |  | Participant age | -0.13(0.03)*** |
|  |  | Female | -0.04(0.03) |
|  |  | Living with a partner | -0.07(0.03)* |
|  | Not being ill | Income level | 0.02(0.04) |
|  |  | Educational level | 0.08(0.03)** |
|  |  | Paid employment | 0.02(0.03) |
|  |  | Participant age | 0.08(0.03)** |
|  |  | Female | 0.02(0.03) |
|  |  | Living with a partner | -0.00(0.03) |
|  |  | Severity of daily hassles | -0.07(0.03)** |
| Model 3b: Sequential mediation:  Severity of daily hassles > a long life  Chi-square (degrees of freedom=1)=65.48, P=0.00  Comparative Fit Index =0.95  Tucker-Lewis Index=0.00  Root Mean Square Error of Approximation=0.22  Standardized Root Mean Square Residual=0.03 | Fruit and vegetable  consumption | Income level | 0.07(0.04) |
|  |  | Educational level | 0.20(0.04)*** |
|  |  | Paid employment | 0.01(0.04) |
|  |  | Participant age | 0.11(0.04)** |
|  |  | Female | 0.08(0.03)* |
|  |  | Living with a partner | -0.01(0.04) |
|  |  | Severity of daily hassles | -0.09(0.03)** |
|  |  | A long life | 0.05(0.03) |
|  | Severity of daily | Income level | -0.22(0.04)*** |
|  | hassles | Educational level | -0.02(0.03) |
|  |  | Paid employment | 0.01(0.03) |
|  |  | Participant age | -0.13(0.03)*** |
|  |  | Female | -0.04(0.03) |
|  |  | Living with a partner | -0.07(0.03)* |
|  | A long life | Income level | 0.03(0.04) |
|  |  | Educational level | -0.10(0.03)** |
|  |  | Paid employment | 0.10(0.03)** |
|  |  | Participant age | 0.02(0.03) |
|  |  | Female | -0.02(0.03) |
|  |  | Living with a partner | 0.08(0.03)* |
|  |  | Severity of daily hassles | -0.04(0.03) |

* p<0.05, ** p<0.01, *** p < 0.001.

Table 3: Sequential mediation SEM coefficients for the outcome transformed snack consumption

| Model: | Outcome: | Predictors: | Imputed estimates (Standard Error): |
| --- | --- | --- | --- |
| Model 3a: Sequential mediation:  Severity of daily hassles > not being ill Chi-square (degrees of freedom=1)=65.37, P=0.00 Comparative Fit Index =0.95 Tucker-Lewis Index=0.00 Root Mean Square Error of Approximation=0.22 Standardized Root Mean Square Residual=0.03 | Snack consumption | Income level | -0.05(0.05) |
|  |  | Educational level | 0.00(0.04) |
|  |  | Paid employment | 0.07(0.04) |
|  |  | Participant age | -0.02(0.03) |
|  |  | Female | -0.02(0.03) |
|  |  | Living with a partner | 0.02(0.04) |
|  |  | Severity of daily hassles | 0.04(0.03) |
|  |  | Not being ill | 0.02(0.03) |
|  | Severity of daily | Income level | -0.22(0.04)*** |
|  | hassles | Education | -0.02(0.03) |
|  |  | Employment | 0.01(0.03) |
|  |  | Participant age | -0.13(0.03)*** |
|  |  | Female | -0.04(0.03) |
|  |  | Living with a partner | -0.07(0.03)* |
|  | Not being ill | Income level | 0.02(0.04) |
|  |  | Educational level | 0.08(0.03)** |
|  |  | Paid employment | 0.02(0.03) |
|  |  | Participant age | 0.08(0.03)** |
|  |  | Female | 0.02(0.03) |
|  |  | Living with a partner | -0.00(0.03) |
|  |  | Severity of daily hassles | -0.07(0.03)** |
| Model 3b: Sequential mediation:  Severity of daily hassles > a long life  Chi-square (degrees of freedom=1)=65.38, P=0.00  Comparative Fit Index =0.95  Tucker-Lewis Index=0.00  Root Mean Square Error of Approximation=0.22  Standardized Root Mean Square Residual=0.03 | Snack  consumption | Income level | -0.05(0.05) |
|  |  | Educational level | 0.00(0.04) |
|  |  | Paid employment | 0.07(0.04) |
|  |  | Participant age | -0.02(0.03) |
|  |  | Female | -0.02(0.03) |
|  |  | Living with a partner | 0.02(0.04) |
|  |  | Severity of daily hassles | 0.04(0.03) |
|  |  | A long life | 0.01(0.03) |
|  | Severity of daily | Income level | -0.22(0.04)*** |
|  | hassles | Educational level | -0.02(0.03) |
|  |  | Paid employment | 0.01(0.03) |
|  |  | Participant age | -0.13(0.03)*** |
|  |  | Female | -0.04(0.03) |
|  |  | Living with a partner | -0.07(0.03)* |
|  | A long life | Income level | 0.03(0.04) |
|  |  | Educational level | -0.10(0.03)** |
|  |  | Paid employment | 0.10(0.03)** |
|  |  | Participant age | 0.02(0.03) |
|  |  | Female | -0.02(0.03) |
|  |  | Living with a partner | 0.08(0.03)* |
|  |  | Severity of daily hassles | -0.04(0.03) |

* p<0.05, ** p<0.01, *** p < 0.001.
