# Supplementary material for: Socioeconomic inequalities in self-assessed health and food consumption: the mediating roles of daily hassles and the perceived importance of health
Source: BMC Public Health. 2023 Mar 7;23:439. doi: 10.1186/s12889-023-15077-0 (PMC9990278; doi:10.1186/s12889-023-15077-0)
Supplement: Supplementary file 4 — Additional file 4. [file 12889_2023_15077_MOESM4_ESM.docx]

**Additional file 4: Full overview of direct, indirect, and total effects estimated in the mediation models.**

Table 1: Overview of direct, indirect, and total effects for single mediation models for self-assessed health

| **Single mediation models for outcome: self-assessed health** | | |  |  |  | **Model fit statistics** | |
| --- | --- | --- | --- | --- | --- | --- | --- |
| **SEP indicator:** | **Mediator:** | | **Indirect effect** | **Direct effect** | **Total effect** | Comparative fit index | Root mean square error of approximation |
| Income | Severity of daily hassles | | 0.04*** | 0.02 | 0.06* | 0.96 | 0.22 |
| Education | Severity of daily hassles | | 0.00 | 0.06* | 0.07** | 0.96 | 0.22 |
| Income | Perceived importance of not being ill | | 0.00 | 0.06* | 0.06* | 0.96 | 0.22 |
| Education | Perceived importance of not being ill | | 0.01*** | 0.06* | 0.07** | 0.96 | 0.22 |
| Income | Perceived importance of living a long life | | 0.00 | 0.06 | 0.06* | 0.96 | 0.22 |
| Education | Perceived importance of living a long life |  | -0.01*** | 0.07** | 0.07** | 0.96 | 0.22 |

Reported effects are statistically significant at *α=0.1, **α=0.05, ***α=0.01.

Table 2: Overview of direct, indirect, and total effects for sequential mediation models for self-assessed health

| **Sequential mediation models for outcome: self-assessed health** | |  |  |  | **Model fit statistics** | |
| --- | --- | --- | --- | --- | --- | --- |
| **SEP indicator:** | **Mediator:** | **Indirect effect** | **Direct effect** | **Total effect** | Comparative fit index | Root mean square error of approximation |
| Income | Severity of daily hassles 🡪 Not being ill | 0.00 | 0.02 | 0.06* | 0.96 | 0.22 |
| Education | Severity of daily hassles 🡪 Not being ill | 0.00 | 0.05* | 0.07** | 0.96 | 0.22 |
| Income | Severity of daily hassles 🡪 Living a long life | 0.00 | 0.02 | 0.06* | 0.96 | 0.22 |
| Education | Severity of daily hassles 🡪 Living a long life | 0.00 | 0.07** | 0.07** | 0.96 | 0.22 |

Reported effects are statistically significant at *α=0.1, **α=0.05, ***α=0.01.

Table 3: Overview of direct, indirect, and total effects for single mediation models for fruit and vegetable consumption

| **Single mediation models for outcome: fruit and vegetable consumption** | | | | | | **Model fit statistics** | |
| --- | --- | --- | --- | --- | --- | --- | --- |
| **SEP indicator:** | **Mediator:** | | **Indirect effect** | **Direct effect** | **Total effect** | Comparative fit index | Root mean square error of approximation |
| Income | Severity of daily hassles | | 0.02** | 0.07 | 0.09* | 0.95 | 0.22 |
| Education | Severity of daily hassles | | 0.00 | 0.20*** | 0.21*** | 0.95 | 0.22 |
| Income | Perceived importance of not being ill | | 0.00 | 0.09* | 0.09* | 0.95 | 0.22 |
| Education | Perceived importance of not being ill | | 0.00 | 0.19*** | 0.21*** | 0.95 | 0.22 |
| Income | Perceived importance of living a long life | | 0.00 | 0.09* | 0.09** | 0.95 | 0.22 |
| Education | Perceived importance of living a long life |  | -0.01 | 0.20*** | 0.21*** | 0.95 | 0.22 |

Reported effects are statistically significant at *α=0.1, **α=0.05, ***α=0.01. For fruit and vegetable consumption, statistical significance was not based on 10,000 bootstrapped samples, but on the average of the 20 imputed datasets.

Table 4: Overview of direct, indirect, and total effects for sequential mediation models for fruit and vegetable consumption

| **Sequential mediation models for outcome: fruit and vegetable consumption** | | | | | **Model fit statistics** | |
| --- | --- | --- | --- | --- | --- | --- |
| **SEP indicator:** | **Mediator:** | **Indirect effect** | **Direct effect** | **Total effect** | Comparative fit index | Root mean square error of approximation |
| Income | Severity of daily hassles 🡪 Not being ill | 0.00 | 0.07 | 0.09* | 0.95 | 0.22 |
| Education | Severity of daily hassles 🡪 Not being ill | 0.00 | 0.19*** | 0.21*** | 0.95 | 0.22 |
| Income | Severity of daily hassles 🡪 Living a long life | 0.00 | 0.07 | 0.09* | 0.95 | 0.22 |
| Education | Severity of daily hassles 🡪 Living a long life | 0.00 | 0.20*** | 0.21*** | 0.95 | 0.22 |

Reported effects are statistically significant at *α=0.1, **α=0.05, ***α=0.01. For fruit and vegetable consumption, statistical significance was not based on 10,000 bootstrapped samples, but on the average of the 20 imputed datasets.

Table 5: Overview of direct, indirect, and total effects for single mediation models for snack consumption

| **Single mediation models for outcome: transformed snack consumption** | | | | | | **Model fit statistics** | |
| --- | --- | --- | --- | --- | --- | --- | --- |
| **SEP indicator:** | **Mediator:** | | **Indirect effect** | **Direct effect** | **Total effect** | Comparative fit index | Root mean square error of approximation |
| Income | Severity of daily hassles | | -0.01 | -0.05 | -0.06 | 0.95 | 0.22 |
| Education | Severity of daily hassles | | -0.00 | -0.01 | -0.01 | 0.95 | 0.22 |
| Income | Perceived importance of not being ill | | 0.00 | -0.06 | -0.06 | 0.95 | 0.22 |
| Education | Perceived importance of not being ill | | 0.00 | 0.00 | -0.01 | 0.95 | 0.22 |
| Income | Perceived importance of living a long life | | 0.00 | -0.06 | -0.06 | 0.95 | 0.22 |
| Education | Perceived importance of living a long life |  | 0.00 | 0.00 | -0.01 | 0.95 | 0.22 |

Reported effects are statistically significant at *α=0.1, **α=0.05, ***α=0.01. For snack consumption, statistical significance was not based on 10,000 bootstrapped samples, but on the average of the 20 imputed datasets.

Table 6: Overview of direct, indirect, and total effects for sequential mediation models for snack consumption

| **Sequential mediation models for outcome: transformed snack consumption** | | | | | **Model fit statistics** | |
| --- | --- | --- | --- | --- | --- | --- |
| **SEP indicator:** | **Mediator:** | **Indirect effect** | **Direct effect** | **Total effect** | Comparative fit index | Root mean square error of approximation |
| Income | Severity of daily hassles 🡪 Not being ill | 0.00 | -0.05 | -0.06 | 0.95 | 0.22 |
| Education | Severity of daily hassles 🡪 Not being ill | 0.00 | 0.00 | -0.01 | 0.95 | 0.22 |
| Income | Severity of daily hassles 🡪 Living a long life | 0.00 | -0.05 | -0.06 | 0.95 | 0.22 |
| Education | Severity of daily hassles 🡪 Living a long life | 0.00 | 0.00 | -0.01 | 0.95 | 0.22 |

Reported effects are statistically significant at *α=0.1, **α=0.05, ***α=0.01. For snack consumption, statistical significance was not based on 10,000 bootstrapped samples, but on the average of the 20 imputed datasets.
