# Supplementary material for: Socioeconomic inequalities in self-assessed health and food consumption: the mediating roles of daily hassles and the perceived importance of health
Source: BMC Public Health. 2023 Mar 7;23:439. doi: 10.1186/s12889-023-15077-0 (PMC9990278; doi:10.1186/s12889-023-15077-0)
Supplement: Supplementary file 5 — Additional file 5. [file 12889_2023_15077_MOESM5_ESM.docx]

**Additional file 5: Mediation results for separate daily hassles.**

Table 1: Standardized indirect effects for mediation models with individual hassles in relation to self-assessed health

| **Standardized indirect effects** | **X = Income level** | **X = Educational level** |
| --- | --- | --- |
| X 🡪 **Legal hassles** 🡪Self-assessed Health | 0.01** | 0.00 |
| X 🡪**Household hassles** 🡪 Self-assessed Health | 0.02** | 0.00 |
| X 🡪 **Organization hassles** 🡪 Self-assessed Health | 0.02** | 0.00 |
| X 🡪 **Paperwork hassles** 🡪 Self-assessed Health | 0.03*** | 0.00 |
| X 🡪 **Home maintenance hassles** 🡪 Self-assessed Health | 0.01*** | -0.00 |
| X 🡪 **Work stress hassles** 🡪 Self-assessed Health | 0.01** | -0.01*** |
| X 🡪 **Job security hassles** 🡪 Self-assessed Health | 0.02*** | -0.00 |
| X 🡪 **Noise pollution hassles** 🡪 Self-assessed Health | 0.02*** | 0.01* |
| X 🡪 **Personal safety hassles** 🡪 Self-assessed Health | 0.02*** | 0.01*** |
| X 🡪 **Discrimination hassles** 🡪 Self-assessed Health | 0.01*** | 0.01*** |
| Total effect | 0.06* | 0.07* |

* p<0.10, ** p<0.05, *** p < 0.01.

Table 2: Standardized indirect effects for mediation models with individual hassles in relation to fruit and vegetable consumption

| **Standardized indirect effects** | **X = Income level** | **X = Educational level** |  |
| --- | --- | --- | --- |
| X 🡪 **Legal hassles** 🡪 Fruit and vegetable consumption | 0.01 | 0.00 |  |
| X 🡪 **Household hassles** 🡪 Fruit and vegetable consumption | 0.01* | 0.00 |  |
| X 🡪 **Organization hassles** 🡪 Fruit and vegetable consumption | 0.01 | 0.00 |  |
| X 🡪 **Paperwork hassles** 🡪 Fruit and vegetable consumption | 0.02** | 0.00 |  |
| X 🡪 **Home maintenance hassles** 🡪 Fruit and vegetable consumption | 0.00 | -0.00 |  |
| X 🡪 **Work stress hassles** 🡪 Fruit and vegetable consumption | 0.00 | -0.00 |  |
| X 🡪 **Job security hassles** 🡪 Fruit and vegetable consumption | 0.01 | 0.00 |  |
| X 🡪 **Noise pollution hassles** 🡪 Fruit and vegetable consumption | -0.00 | -0.00 |  |
| X 🡪 **Personal safety hassles** 🡪 Fruit and vegetable consumption | 0.01* | 0.01 |  |
| X 🡪 **Discrimination hassles** 🡪 Fruit and vegetable consumption | 0.01 | 0.00 |  |
| Total effect | 0.09** | 0.21*** |  |

* p<0.10, ** p<0.05, *** p < 0.01.

Table 3: Standardized indirect effects for mediation models with individual hassles in relation to snack consumption

| **Standardized indirect effects** | **X = Income level** | **X = Educational level** |  |
| --- | --- | --- | --- |
| X 🡪 **Legal hassles** 🡪 Snack consumption | -0.01 | -0.00 |  |
| X 🡪 **Household hassles** 🡪 Snack consumption | -0.00 | 0.00 |  |
| X 🡪 **Organization hassles** 🡪 Snack consumption | -0.01 | 0.00 |  |
| X 🡪 **Paperwork hassles** 🡪 Snack consumption | -0.00 | 0.00 |  |
| X 🡪 **Home maintenance hassles** 🡪 Snack consumption | -0.01 | 0.00 |  |
| X 🡪 **Work stress hassles** 🡪 Snack consumption | -0.00 | 0.00 |  |
| X 🡪 **Job security hassles** 🡪 Snack consumption | 0.01 | 0.00 |  |
| X 🡪 **Noise pollution hassles** 🡪 Snack consumption | 0.00 | 0.00 |  |
| X 🡪 **Personal safety hassles** 🡪 Snack consumption | -0.00 | -0.00 |  |
| X 🡪 **Discrimination hassles** 🡪 Snack consumption | 0.00 | 0.00 |  |
| Total effect | -0.09** | -0.03 |  |

* p<0.10, ** p<0.05, *** p < 0.01.
