# Supplementary material for: Socioeconomic inequalities in self-assessed health and food consumption: the mediating roles of daily hassles and the perceived importance of health
Source: BMC Public Health. 2023 Mar 7;23:439. doi: 10.1186/s12889-023-15077-0 (PMC9990278; doi:10.1186/s12889-023-15077-0)
Supplement: Supplementary file 6 — Additional file 6. [file 12889_2023_15077_MOESM6_ESM.docx]

**Additional file 6: Results for (alternative) moderated mediation hypothesis.**

The next Figures visualize the relevant pathways in relation to the moderated mediation hypothesis. The model includes the covariates: age, gender, living with a partner, and employment, yet the coefficients are excluded from this visualization. These models were run using PROCESS MACRO in SPSS (model 14), and all estimates were bootstrapped 10,000 times.


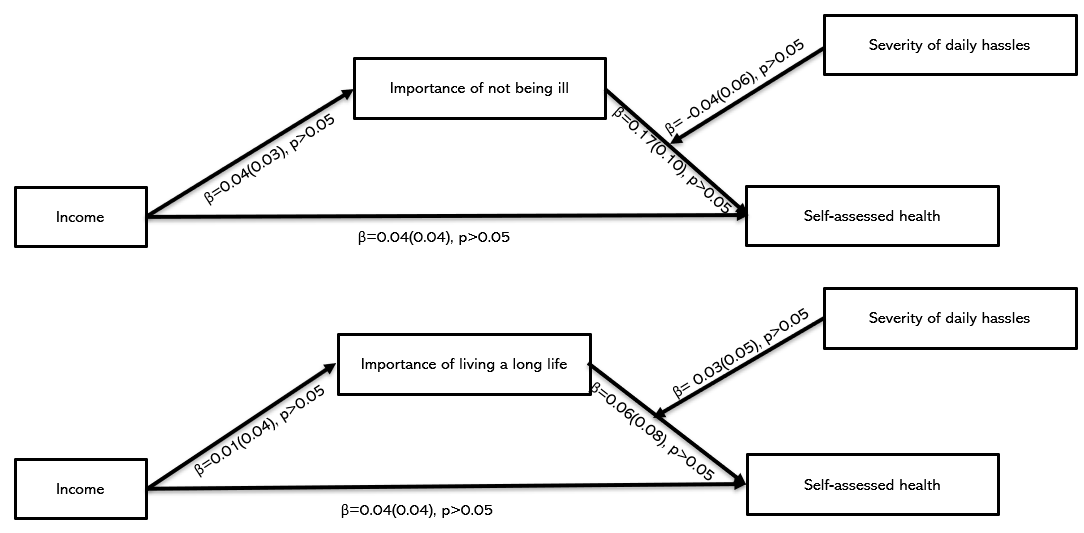


Figure 1: Moderated mediation of daily hassles and the perceived importance of health (perceived importance of not being ill on top, perceived importance of living a long life on the bottom), on the association between income level and self-assessed health.

Figure 1 indicates that there is no evidence of a moderated mediation of daily hassles on the association between the perceived importance of health (operationalized as either the perceived importance of not being ill or the perceived importance of living a long life) and self-assessed health.


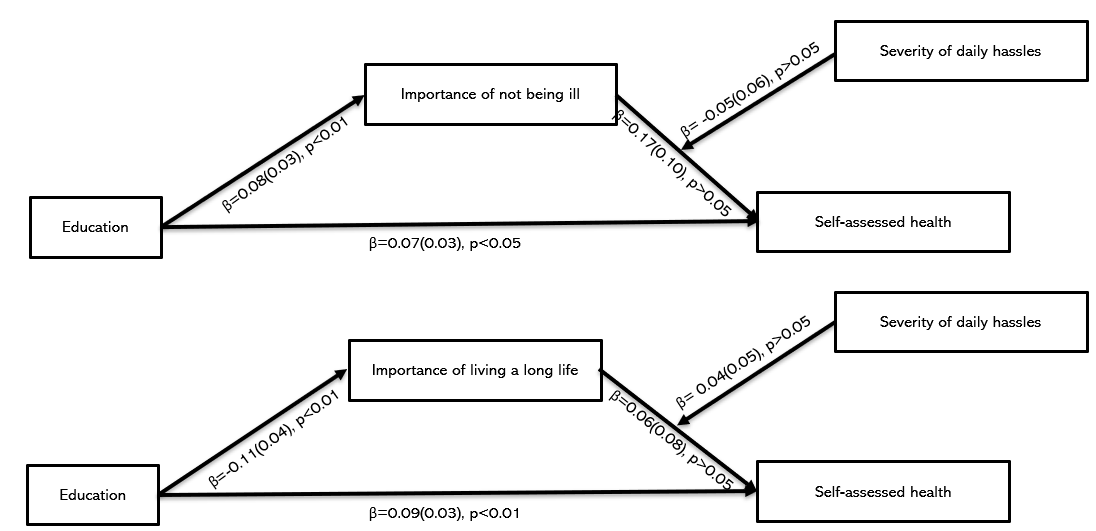


Figure 2: Moderated mediation of daily hassles and the perceived importance of health (perceived importance of not being ill on top, perceived importance of living a long life on the bottom), on the association between educational level and self-assessed health.

Figure 2 indicates that there is no evidence of a moderated mediation of daily hassles on the association between the perceived importance of health (operationalized as either the perceived importance of not being ill or the perceived importance of living a long life) and self-assessed health.


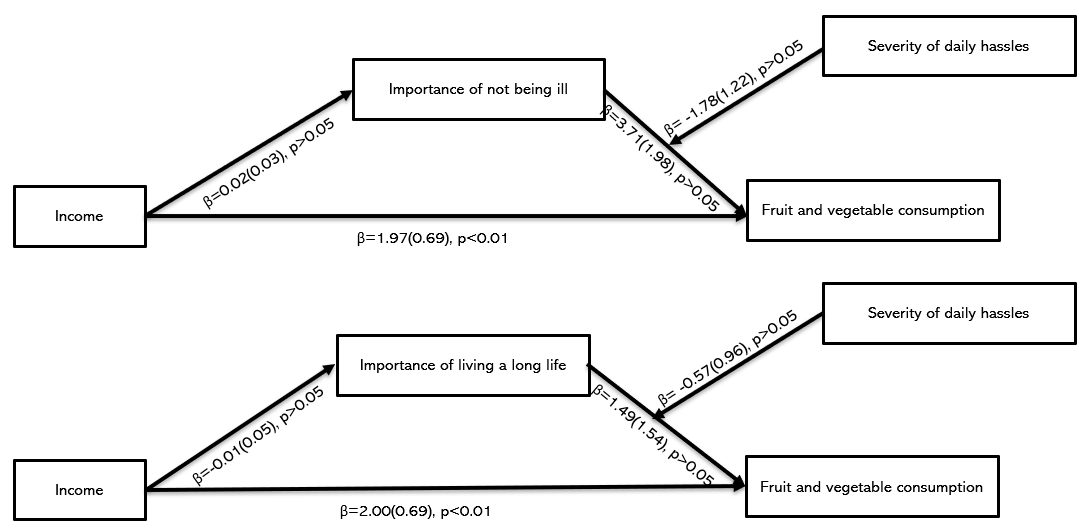


Figure 3: Moderated mediation of daily hassles and the perceived importance of health (perceived importance of not being ill on top, perceived importance of living a long life on the bottom), on the association between income level and fruit and vegetable consumption.

Figure 3 indicates that there is no evidence of a moderated mediation of daily hassles on the association between the perceived importance of health (operationalized as either the perceived importance of not being ill or the perceived importance of living a long life) and fruit and vegetable consumption. Note that the Fruit and vegetable consumption outcome was based on non-imputed data (n=1051).


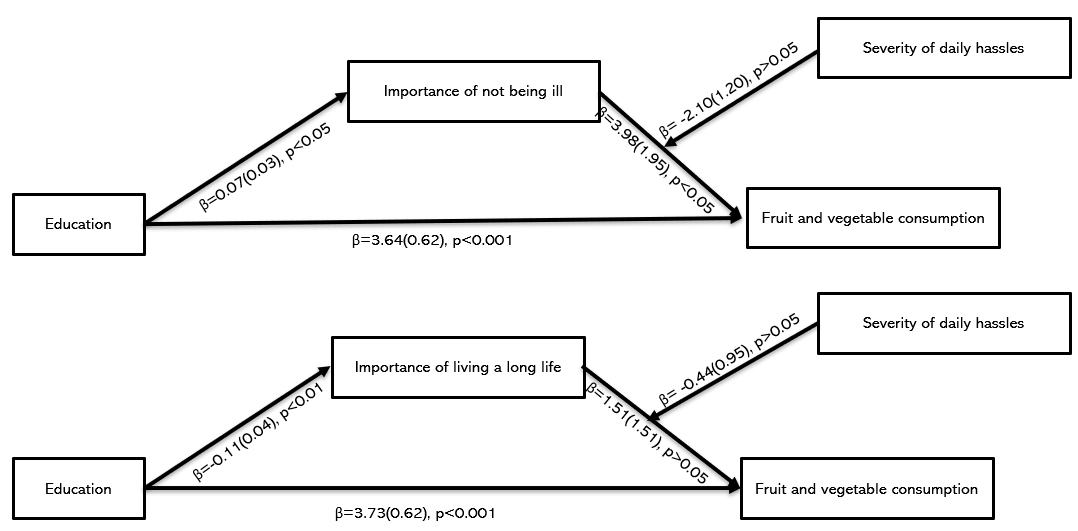


Figure 4: Moderated mediation of daily hassles and the perceived importance of health (perceived importance of not being ill on top, perceived importance of living a long life on the bottom), on the association between educational level and fruit and vegetable consumption.

Figure 4 indicates that there is no evidence of a moderated mediation of daily hassles on the association between the perceived importance of health (operationalized as either the perceived importance of not being ill or the perceived importance of living a long life) and fruit and vegetable consumption. Note that the Fruit and vegetable consumption outcome was based on non-imputed data (n=1051).


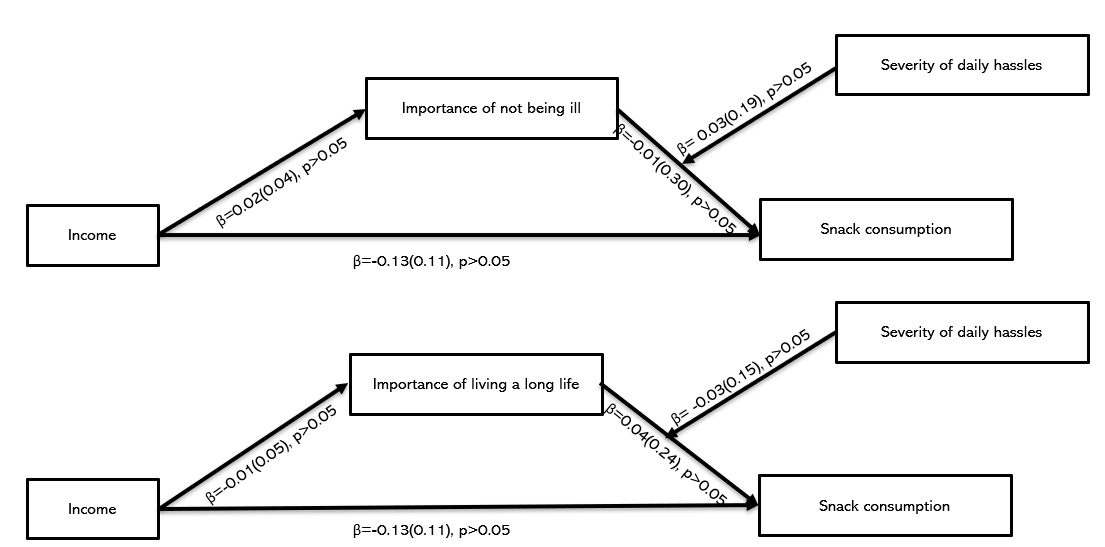


Figure 5: Moderated mediation of daily hassles and the perceived importance of health (perceived importance of not being ill on top, perceived importance of living a long life on the bottom), on the association between income level and snack consumption.

Figure 5 indicates that there is no evidence of a moderated mediation of daily hassles on the association between the perceived importance of health (operationalized as either the perceived importance of not being ill or the perceived importance of living a long life) and snack consumption. Note that the snack consumption outcome was based on the square root transformation of non-imputed data (n=1051).


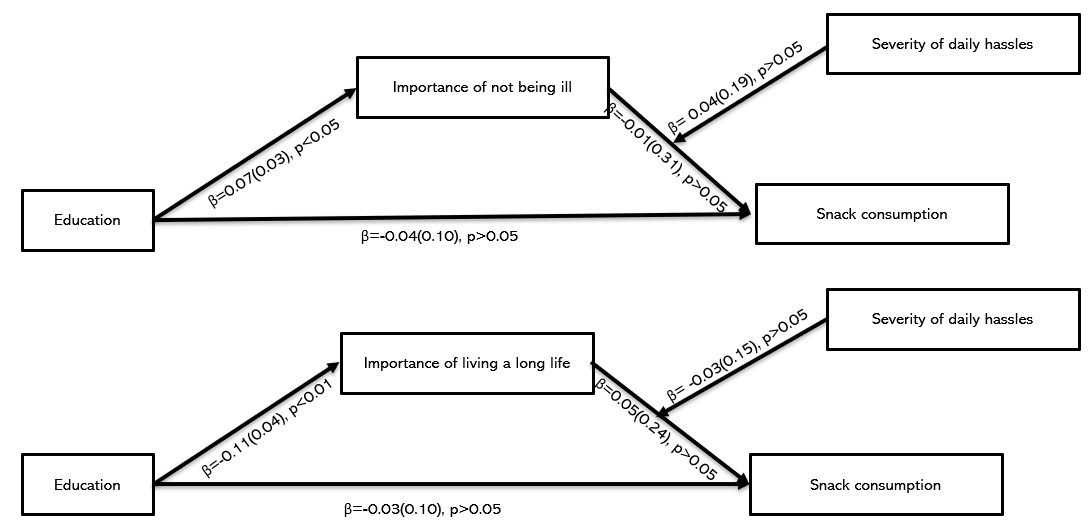


Figure 6: Moderated mediation of daily hassles and the perceived importance of health (perceived importance of not being ill on top, perceived importance of living a long life on the bottom), on the association between educational level and snack consumption.

Figure 5 indicates that there is no evidence of a moderated mediation of daily hassles on the association between the perceived importance of health (operationalized as either the perceived importance of not being ill or the perceived importance of living a long life) and snack consumption. Note that the snack consumption outcome was based on the square root transformation of non-imputed data (n=1051).
