# Supplementary material for: Socioeconomic inequalities in self-assessed health and food consumption: the mediating roles of daily hassles and the perceived importance of health
Source: BMC Public Health. 2023 Mar 7;23:439. doi: 10.1186/s12889-023-15077-0 (PMC9990278; doi:10.1186/s12889-023-15077-0)
Supplement: Supplementary file 7 — Additional file 7. [file 12889_2023_15077_MOESM7_ESM.docx]

**Additional file 7: Structural equation model coefficients for untransformed snack consumption data.**

Table 1: Standardized coefficients of structural equation model with untransformed outcome snack consumption.

| Model: | Outcome: | Predictors: | Imputed estimates (Standard Error): |
| --- | --- | --- | --- |
| Model 1= Baseline  Chi-square (degrees of freedom=1)=65.35, p=0.00  Comparative Fit Index=0.94 Tucker-Lewis Index=0.12 Root Mean Square Error of Approximation=0.22 Standardized Root Mean Square Residual=0.04 | Snack consumption | Income level | -0.09(0.05) |
|  |  | Educational level | -0.01(0.04) |
|  |  | In paid employment | 0.04(0.04) |
|  |  | Participant age | -0.01(0.04) |
|  |  | Female | -0.03(0.03) |
|  |  | Living with a partner | 0.01(0.04) |
| Model 2A=Model 1 + Mediator Severity of daily hassles  Chi-square (degrees of freedom=1)=65.36, p=0.00 Comparative Fit Index=0.95  Tucker-Lewis Index=0.00 Root Mean Square Error of Approximation=0.22 | Snack consumption | Income level | -0.08(0.04) |
|  |  | Educational level | -0.01(0.04) |
|  |  | In paid employment | 0.04(0.04) |
|  |  | Participant age | 0.01(0.04) |
|  |  | Female | -0.03(0.03) |
|  |  | Living with a partner | 0.02(0.04) |
| Standardized Root Mean Square Residual=0.04 |  | Severity of daily hassles | 0.03(0.03) |
|  | Severity of daily hassles | Income level | -0.22(0.04)*** |
|  |  | Educational level | -0.02(0.03) |
|  |  | In paid employment | 0.01(0.03) |
|  |  | Participant age | -0.13(0.03)*** |
|  |  | Female | -0.04(0.03) |
|  |  | Living with a partner | -0.07(0.03)* |
| Model 2B=Model 1 + Mediator not being ill  Chi-square (degrees of freedom=1)=65.34, p=0.00  Comparative Fit Index=0.94 Tucker-Lewis Index=0.00 Root Mean Square Error of Approximation=0.22  Standardized Root Mean Square Residual=0.04 | Snack consumption | Income level | -0.09(0.05) |
|  |  | Educational level | -0.02(0.04) |
|  |  | In paid employment | 0.04(0.04) |
|  |  | Participant age | 0.01(0.04) |
|  |  | Female | -0.03(0.03) |
|  |  | Living with a partner | 0.01(0.04) |
|  |  | Not being ill | 0.01(0.03) |
|  | Not being ill | Income level | 0.03(0.04) |
|  |  | Educational level | 0.08(0.03)** |
|  |  | In paid employment | 0.02(0.03) |
|  |  | Participant age | 0.09(0.03)** |
|  |  | Female | 0.02(0.03) |
|  |  | Living with a partner | 0.01(0.03) |
| Model 2C=model 1 + Mediator Living a long life  Chi-square (degrees of freedom=1)=65.34, p=0.00,  Comparative Fit Index=0.95 Tucker-Lewis Index=0.00 Root Mean Square Error of Approximation=0.22  Standardized Root Mean Square Residual=0.04 | Snack consumption | Income level | -0.09(0.05) |
|  |  | Educational level | -0.01(0.04) |
|  |  | In paid employment | 0.04(0.04) |
|  |  | Participant age | 0.01(0.04) |
|  |  | Female | -0.03(0.03) |
|  |  | Living with a partner | 0.01(0.04) |
|  |  | A long life | -0.00(0.03) |
|  | A long life | Income level | 0.04(0.04) |
|  |  | Educational level | -0.10(0.03)** |
|  |  | In paid employment | 0.10(0.03)** |
|  |  | Participant age | 0.03(0.03) |
|  |  | Female | -0.02(0.03) |
|  |  | Living with a partner | 0.08(0.03)* |

* p<0.05, ** p<0.01, *** p < 0.001.

Table 2: Sequential mediation SEM coefficients for the outcome untransformed snack consumption

| Model: | Outcome: | Predictors: | Imputed estimates (Standard Error): |
| --- | --- | --- | --- |
| Model 3a: Sequential mediation:  Severity of daily hassles > not being ill Chi-square (degrees of freedom=1)=65.35, P=0.00 Comparative Fit Index =0.95 Tucker-Lewis Index=0.00 Root Mean Square Error of Approximation=0.22 Standardized Root Mean Square Residual=0.03 | Snack consumption | Income level | -0.08(0.04) |
|  |  | Educational level | -0.01(0.04) |
|  |  | Paid employment | 0.05(0.04) |
|  |  | Participant age | 0.01(0.03) |
|  |  | Female | -0.03(0.03) |
|  |  | Living with a partner | 0.02(0.04) |
|  |  | Severity of daily hassles | 0.03 (0.03) |
|  |  | Not being ill | 0.01(0.03) |
|  | Severity of daily | Income level | -0.22(0.04)*** |
|  | hassles | Education | -0.02(0.03) |
|  |  | Employment | 0.01(0.03) |
|  |  | Participant age | -0.13(0.03)*** |
|  |  | Female | -0.04(0.03) |
|  |  | Living with a partner | -0.07(0.03)* |
|  | Not being ill | Income level | 0.02(0.04) |
|  |  | Educational level | 0.08(0.03)** |
|  |  | Paid employment | 0.02(0.03) |
|  |  | Participant age | 0.08(0.03)** |
|  |  | Female | 0.02(0.03) |
|  |  | Living with a partner | -0.00(0.03) |
|  |  | Severity of daily hassles | -0.07(0.03)** |
| Model 3b: Sequential mediation:  Severity of daily hassles > a long life  Chi-square (degrees of freedom=1)=65.35, P=0.00  Comparative Fit Index =0.95  Tucker-Lewis Index=0.00  Root Mean Square Error of Approximation=0.22  Standardized Root Mean Square Residual=0.03 | Snack  consumption | Income level | -0.08(0.04) |
|  |  | Educational level | -0.01(0.04) |
|  |  | Paid employment | 0.04(0.04) |
|  |  | Participant age | 0.01(0.03) |
|  |  | Female | -0.03(0.03) |
|  |  | Living with a partner | 0.02(0.04) |
|  |  | Severity of daily hassles | 0.03(0.03) |
|  |  | A long life | 0.01(0.03) |
|  | Severity of daily | Income level | -0.22(0.04)*** |
|  | hassles | Educational level | -0.02(0.03) |
|  |  | Paid employment | 0.01(0.03) |
|  |  | Participant age | -0.13(0.03)*** |
|  |  | Female | -0.04(0.03) |
|  |  | Living with a partner | -0.07(0.03)* |
|  | A long life | Income level | 0.03(0.04) |
|  |  | Educational level | -0.10(0.03)** |
|  |  | Paid employment | 0.10(0.03)** |
|  |  | Participant age | 0.02(0.03) |
|  |  | Female | -0.02(0.03) |
|  |  | Living with a partner | 0.08(0.03)* |
|  |  | Severity of daily hassles | -0.04(0.03) |

* p<0.05, ** p<0.01, *** p < 0.001.

Table 3: Full overview of direct, indirect, and total effects estimated in the single mediation models.

| **Single mediation models for outcome: untransformed snack consumption** | | | | | | **Model fit statistics** | |
| --- | --- | --- | --- | --- | --- | --- | --- |
| **SEP indicator:** | **Mediator:** | | **Indirect effect** | **Direct effect** | **Total effect** | Comparative fit index | Root mean square error of approximation |
| Income | Severity of daily hassles | | -0.01 | -0.08* | -0.09*** | 0.95 | 0.22 |
| Education | Severity of daily hassles | | -0.00 | -0.01 | -0.03 | 0.95 | 0.22 |
| Income | Perceived importance of not being ill | | 0.00 | -0.09** | -0.09** | 0.94 | 0.22 |
| Education | Perceived importance of not being ill | | 0.00 | -0.02 | -0.03 | 0.94 | 0.22 |
| Income | Perceived importance of living a long life | | 0.00 | -0.09** | -0.09** | 0.95 | 0.22 |
| Education | Perceived importance of living a long life |  | -0.00 | -0.01 | -0.03 | 0.95 | 0.22 |

Reported effects are statistically significant at *α=0.1, **α=0.05, ***α=0.01.

Table 4: Full overview of direct, indirect, and total effects estimated in the sequential mediation models.

| **Sequential mediation models for outcome: untransformed snack consumption** | | | | | **Model fit statistics** | |
| --- | --- | --- | --- | --- | --- | --- |
| **SEP indicator:** | **Mediator:** | **Indirect effect** | **Direct effect** | **Total effect** | Comparative fit index | Root mean square error of approximation |
| Income | Severity of daily hassles 🡪 Not being ill | 0.00 | -0.08* | -0.09** | 0.95 | 0.22 |
| Education | Severity of daily hassles 🡪 Not being ill | 0.00 | -0.01 | -0.03 | 0.95 | 0.22 |
| Income | Severity of daily hassles 🡪 Living a long life | 0.00 | -0.08* | -0.09** | 0.95 | 0.22 |
| Education | Severity of daily hassles 🡪 Living a long life | 0.00 | -0.01 | -0.03 | 0.95 | 0.22 |

Reported effects are statistically significant at *α=0.1, **α=0.05, ***α=0.01.
